# Regenerative medicine

**DOI:** 10.1186/s12967-024-05521-z

**Published:** 2024-08-05

**Authors:** Francisco Silva

**Affiliations:** BioRestorative Therapies, Melville, NY USA

We are pleased to announce the launch of *Regenerative Medicine*, a new section of the Journal of Translational Medicine. This section encourages contributions aimed at advancing the research and development and clinical translation in the emerging field of regenerative medicine.

The clinically disruptive field of regenerative medicine is poised to transform 21st century healthcare, it encompasses various strategies including the use of materials and biologics to take the place or remodel compromised tissues both structurally and functionally in order to facilitate tissue healing. The ability to remodel tissue and organ microenvironments damaged by age, disease, or trauma has a tremendous impact on the current standard of care in treating patients suffering from various diseases.

Over the past two decades tissue engineering and regenerative medicine has emerged as an exciting path towards therapies. A broad range of strategies in both the preclinical and clinical stages of investigation are currently being explored. Key areas to consider for the successful translation are: (i) recapitulating organ and tissue structure and microenvironments using 3D synthetic or biologic based scaffolds, (ii) strategies for vascularization and innervation of grafts are critical for integration within the host, (iii) modulating the host immune system and microenvironment via cell infusion and finally the development and use of novel cell types and sources.

Tissue and organ function is deeply correlated with its respective architecture and microenvironment, the ability to remodel damaged or diseased structure is essential for successful cell and tissue function [1]. The use of decellularized organs and extra cellular matrixes followed by recellularization before transplantation are currently under development or in various stages of clinical use [2]. Synthetic scaffolds derived from algae-derived alginate or from synthetic polymers such as poly(lactide-coglycolide) have also emerged as an alternative option to remodel tissue and organ architecture [3].

Bioengineered grafts in order to function effectively must integrate within the host. Vascularization is critical for essential for nutrient exchange and oxygen diffusion from the native bloodstream to occur [4]. Several strategies have been employed including combing the use of a variety of growth factors that are involved in angiogenesis, such as vascular endothelial growth factor (VEGF), platelet-derived growth factor (PDGF) and basic fibroblast growth factor (bFGF) [5, 6]. In addition to vascularization, innervation is also required for full integration and critical for some grafts to function properly [7, 8] in particular where motor control or sensation is involved such as skeletal muscle or the epidermis [9, 10].

Modulating the host immune system and microenvironment via cell infusion has shown great promise. Application of cells can induce therapeutic responses without the use of 3D grafts by indirect means, such as the secretion of growth factors or extracellular vesicles that interact with host cells and microenvironments [11, 12]. Transplanted cells can remodel injured or diseased tissues, by altering the extracellular matrix or providing a systemic paracrine effect which may initiate a therapeutic response.

Regenerative medicine is based on the use of biologics such as cells, in order to fully optimize therapeutics for various indications, identifying and obtaining sufficient cells is often a challenge. Stem, progenitor or differentiated cells derived from various tissues sources is being explored [13]. The clinical translation of novel cell sources and types such as modified mesenchymal stem cells, embryonic stem cells and induced pluripotent stem cells have advanced considerably in the past decade with several first in man clinical trials demonstrating safety and feasibility completed [14].

The field of regenerative medicine brings together experts in biology, chemistry, computer science, engineering, genetics, medicine, robotics, and other disciplines to find solutions to some of the most challenging medical problems. An improved understanding of how age, environmental factors and the various stages of disease progression and their impact on the innate regeneration will also likely be important in advancing the field.

The launch of the *Regenerative Medicine* section of the Journal of Translational Medicine seeks to provide scientist across the world open access, high-level peer-review process and rapid publication times. The studies reviewed and published are aimed at advancing the various technologies that encompass regenerative medicine which ultimately will aid in the development and translation of novel therapies for those suffering from unmet medical needs. On behalf of the Editorial board, I look forward to receiving your contributions and novel findings to *Regenerative Medicine* section.
